# Internal tsunamigenesis and ocean mixing driven by glacier calving in Antarctica

**DOI:** 10.1126/sciadv.add0720

**Published:** 2022-11-23

**Authors:** Michael P. Meredith, Mark E. Inall, J. Alexander Brearley, Tobias Ehmen, Katy Sheen, David Munday, Alison Cook, Katherine Retallick, Katrien Van Landeghem, Laura Gerrish, Amber Annett, Filipa Carvalho, Rhiannon Jones, Alberto C. Naveira Garabato, Christopher Y. S. Bull, Benjamin J. Wallis, Anna E. Hogg, James Scourse

**Affiliations:** ^1^ British Antarctic Survey, Cambridge, UK.; ^2^ Scottish Association for Marine Science, Oban, UK.; ^3^ University of Exeter, Exeter, UK.; ^4^ Bangor University, Wales, UK.; ^5^ Ocean and Earth Science, University of Southampton, Southampton, UK.; ^6^ National Oceanography Centre, Southampton, UK.; ^7^ Department of Geography and Environmental Sciences, Northumbria University, Newcastle upon Tyne, UK.; ^8^ School of Earth and Environment, University of Leeds, Leeds, UK.

## Abstract

Ocean mixing around Antarctica exerts key influences on glacier dynamics and ice shelf retreats, sea ice, and marine productivity, thus affecting global sea level and climate. The conventional paradigm is that this is dominated by winds, tides, and buoyancy forcing. Direct observations from the Antarctic Peninsula demonstrate that glacier calving triggers internal tsunamis, the breaking of which drives vigorous mixing. Being widespread and frequent, these internal tsunamis are at least comparable to winds, and much more important than tides, in driving regional shelf mixing. They are likely relevant everywhere that marine-terminating glaciers calve, including Greenland and across the Arctic. Calving frequency may change with higher ocean temperatures, suggesting possible shifts to internal tsunamigenesis and mixing in a warming climate.

## Teaser

Tsunamis are generated within the ocean by glacier calving, driving vigorous mixing in areas key for climate and sea level.

## INTRODUCTION

Transformations of Antarctic continental shelf waters exert a global climatic influence (*1*). Heat from these waters drives the retreat of marine-terminating glaciers and ice shelves that fringe Antarctica (*2*–*4*), with consequences for ice sheet stability and sea-level rise (*1*, *5*). Dense water production on Antarctic shelves replenishes the lower limb of the oceanic overturning circulation [i.e., the deepest layer of the three-dimensional (3D) global system of ocean currents], thus exerting a pervasive influence on global climate (*6*, *7*). Antarctic shelf waters are typically highly productive due to the injection of micronutrients from glaciers and sediments (*8*), with both surface glacial melt and subglacial plumes subsidizing primary production (*9*). Vertical mixing of deep waters replete in macronutrients supports the food web locally (*10*) and facilitates drawdown of carbon from the atmosphere (*11*, *12*).

Diverse studies have sought to measure and understand diapycnal mixing (i.e., mixing across density surfaces) on the Antarctic shelves (*13*–*15*). These have led to the conventional paradigm of buoyancy forcing, winds, and tides driving primarily turbulent mixing, i.e., microscale chaotic fluctuations of scalar properties (e.g., pressure, temperature, and salinity) and velocity within seawater that homogenize tracers such as heat, salt, and nutrients (*13*, *16*, *17*). Buoyancy forcing occurs through heat loss and sea ice production in winter; combined with restratification in summer, this sets the seasonal cycle of water column stability. Winds and tides exert a year-round destratifying influence, modulated by sea ice and influenced by topography (*18*). Mixing has been incorporated into ocean and climate models using representations or parameterizations of these processes. In this study, we use data from an Antarctic research expedition, Earth observation satellites, and numerical modeling to demonstrate that, contrary to the conventional paradigm, the generation, propagation, and breaking of internal tsunamis triggered by the calving of marine-terminating glaciers represent a leading-order source of diapycnal mixing.

## RESULTS

### Glacier calving and ocean homogenization

**Fig. 1.**
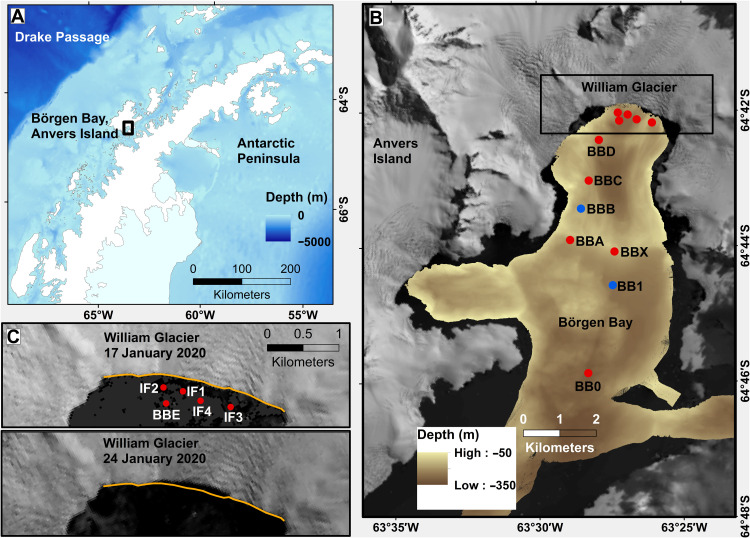
Field area and glacier retreat due to calving. (**A**) Location of Börgen Bay at the West Antarctic Peninsula. Bathymetry from ETOPO 1 global relief model (*74*). (**B**) Bathymetry of Börgen Bay from multibeam echosounder data and coastline/topography from Landsat imagery (see Materials and Methods). Dots mark locations of conductivity-temperature-depth (CTD) profiles used here, with profiles taken before (red) and after (blue) the calving event. (**C**) Landsat images of the William Glacier front from (top) 17 January 2020 and (bottom) 24 January 2020. In both panels, the orange line marks the glacier front on 17 January 2020 to highlight the retreat of the glacier between those dates.

On 21 January 2020, a calving event occurred at the marine terminus of the William Glacier in Börgen Bay, Anvers Island (Fig. 1), a ~300-m-deep embayment at the Antarctic Peninsula. The glacier has a height above sea level of 42 m at its terminus; it is grounded about 210 m below sea level in its northern approach and about 150 m in its western approach (fig. S1), and it does not have a notable floating ice tongue. The flow speed averages around 500 m/year near the ice front (fig. S1). The glacier is ~4.5 km wide, and calving extended across ~1 km of the ice front (Fig. 1C), with ~78,000-m^2^ surface area of ice discharged. The event was characteristic of ice front disintegration, as opposed to calving of a single intact iceberg. The amount of ice discharged below sea level is not known absolutely; however, we quantify the total volume discharged to be 3 to 20 × 10^6^ m^3^, and the potential energy released to the ocean to be 0.6 to 2.4 × 10^12^ J (see Materials and Methods).

**Fig. 2.**
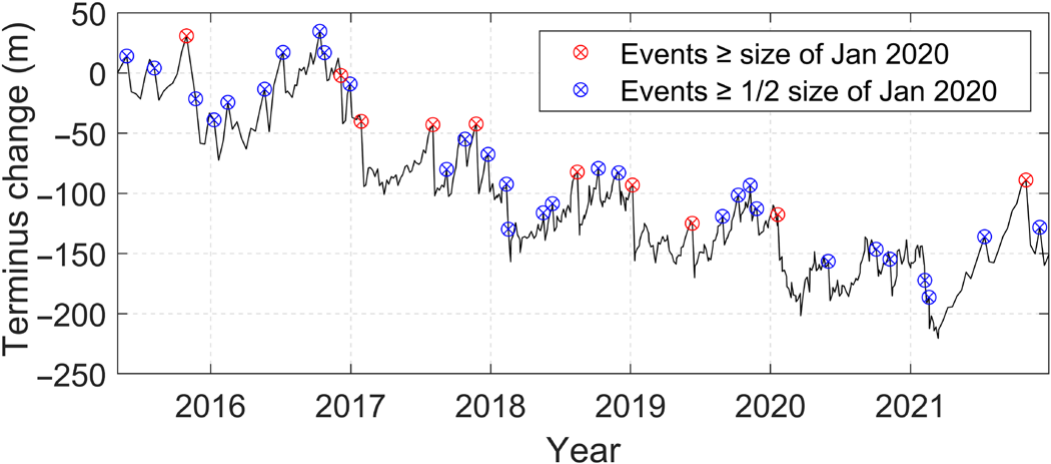
Frequency of calving in Börgen Bay. A time series of the terminus position of William Glacier digitized from Sentinel-1 synthetic aperture radar (SAR) imagery, acquired between 3 May 2015 and 28 December 2021 (see Materials and Methods). This sequence places the 21 January 2020 calving event in the longer-term context of the terminus position change, particularly in relation to the size/frequency of other calving events. In the May 2015 to December 2021 period, we observe nine other terminus retreat events of equivalent or greater magnitude (red symbols) and a total of 39 other events at least half as large (blue symbols). These terminus retreats correspond to large collapses of the ice front and input of solid ice into Börgen Bay, although the calving behavior in the period between satellite images cannot be resolved exactly. Events half as large as January 2020 occur most frequently in November, and there is at least one event larger than January 2020 in every austral summer except 2020 to 2021. Although less frequent, there are also some notable winter retreats, with events larger than January 2020 occurring in August 2017, August 2018, and June 2019. This time series also shows a trend of multiyear retreat through this period, with the terminus of William Glacier retreating by 118 ± 10 m from 29 December 2015 to 28 December 2021.

**Fig. 3.**
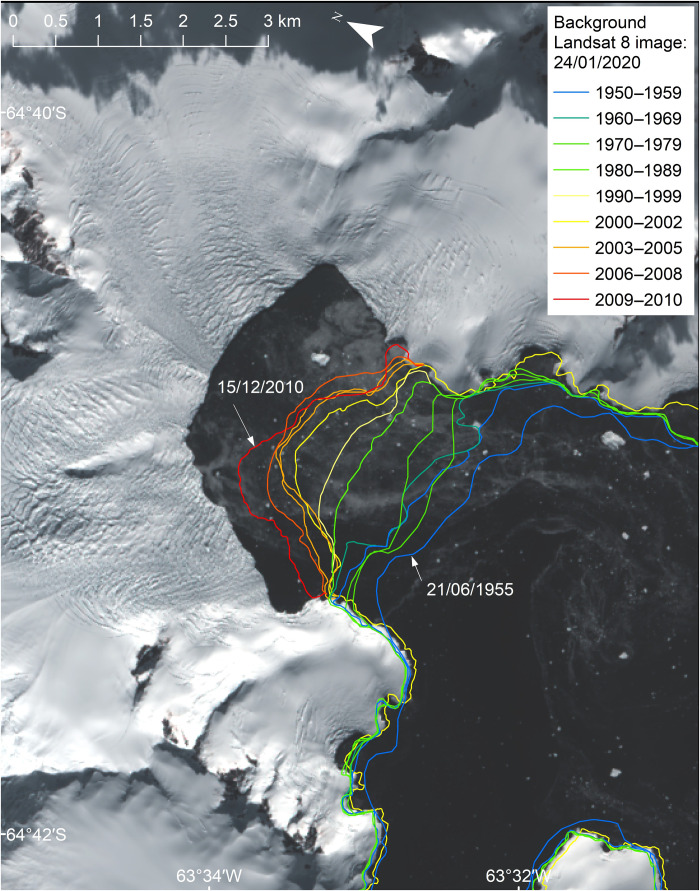
Decadal retreat of William Glacier since the 1950s. Mean retreat has been approximately 55 m/year over the past seven decades. The frontal positions shown were digitized from a range of image sources, including optical satellite imagery and vertical aerial photography. Methods for the procedure used to map glacier fronts around the Antarctic Peninsula are given in Cook *et al.* (*19*).

Calving of this type and scale is frequent and widespread. Satellite monitoring between 2015 and 2021 reveals one to two calving events per year from the William Glacier with the same magnitude or larger as the January 2020 event and approximately six times as many calving events with at least half the magnitude (see Materials and Methods; Fig. 2). The decadal retreat of the glacier, where the calving front has moved landward around 55 m/year since the middle of the last century (Fig. 3), is consistent with ice loss from calving processes exceeding the ice flux to the ice front over the long term. Such retreat is characteristic for the majority of glaciers at the Antarctic Peninsula (*19*).

**Fig. 4.**
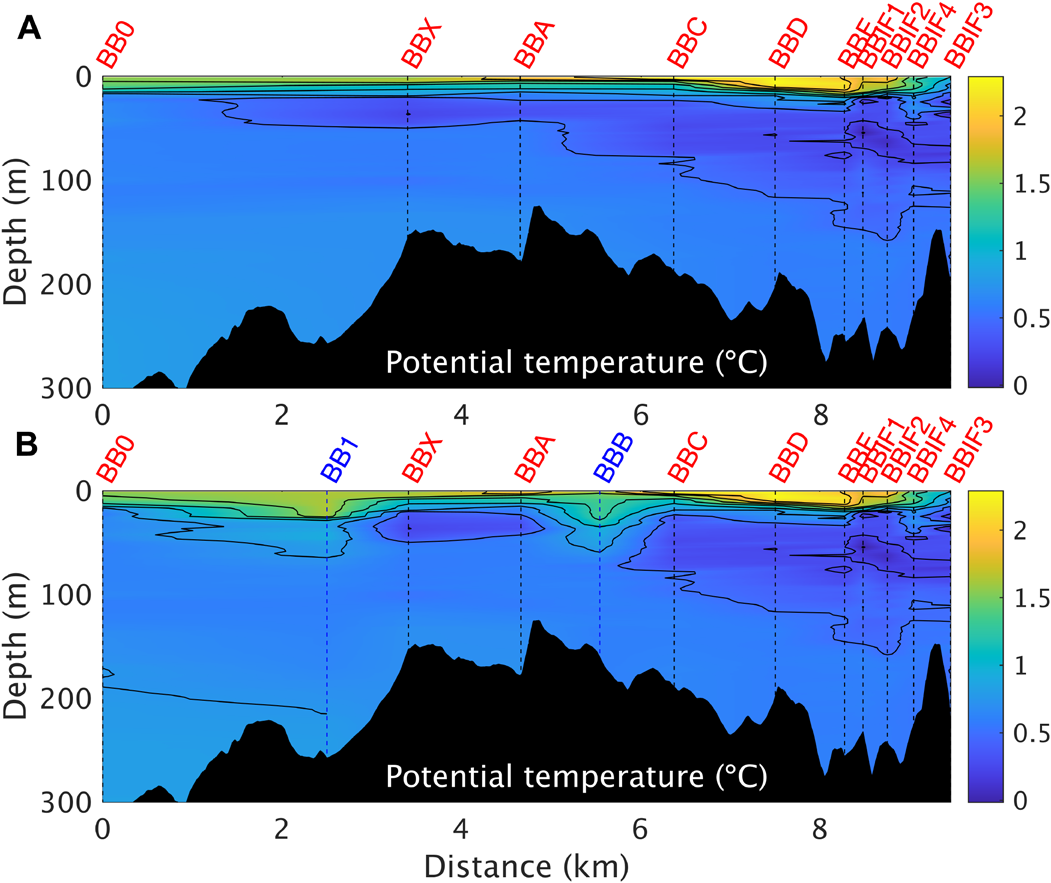
Vertical temperature structure of the ocean before and after the calving event. (**A**) Potential temperature across Börgen Bay using only precalving stations marked in red in Fig. 1B. William Glacier is on the right. (**B**) As per (A) but including also the postcalving stations (BB1 and BBB) labeled in blue.

**Fig. 5.**
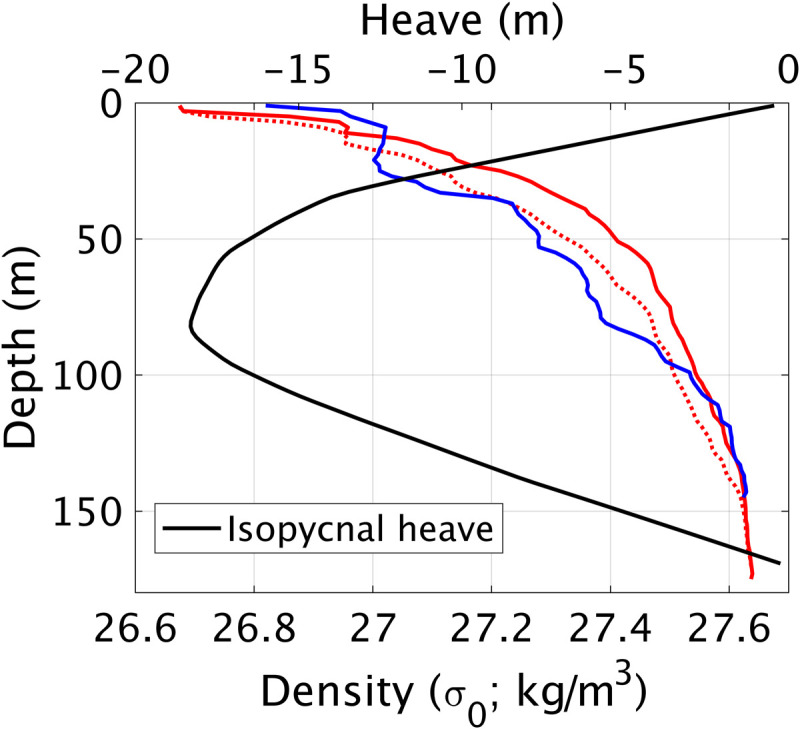
Density profiles before (BBA; red) and after (BBB; blue) the calving event. Solid line is original data, and dashed line is with data adjusted to conserve mass before and after the calving and to account for spatial separation of casts. Black is the amount of heave adjustment applied (see Materials and Methods).

The January 2020 calving event was witnessed directly from RRS *James Clark Ross*, which was undertaking oceanographic measurements in Börgen Bay at the time (see Materials and Methods). Before the calving, Börgen Bay featured a pronounced subsurface temperature minimum at around 50 to 100 m in depth, characteristic of the Southern Ocean in summer but here relating also to the subsurface spreading of glacial meltwater (Fig. 4 and fig. S2). Below this, warmer waters advected from offshore provide heat for the melting of the William Glacier. After the calving, the water column structure differed markedly, being greatly more homogeneous (Figs. 4B and 5).

The destratification can be quantified by the change in a depth-integrated potential energy anomaly ϕ, giving ∆ϕ = 6074 J m^−2^ (see Materials and Methods). That the mixing covered the entirety of Börgen Bay is evidenced by continuous near-surface underway data (figs. S3 and S4). If taken across the full area of Börgen Bay, then ∆ϕ would equate to approximately 2.1 × 10^11^ J. Comparison with the 0.6 to 2.4 × 10^12^ J available from calving indicates that the mixing and homogenization of the water column in Börgen Bay used a moderate proportion (although certainly not the majority) of energy released from the calving event. Further mixing outside Börgen Bay will have occurred but is unquantified here; this would increase this proportion.

### Internal tsunami generation and breaking

**Fig. 6.**
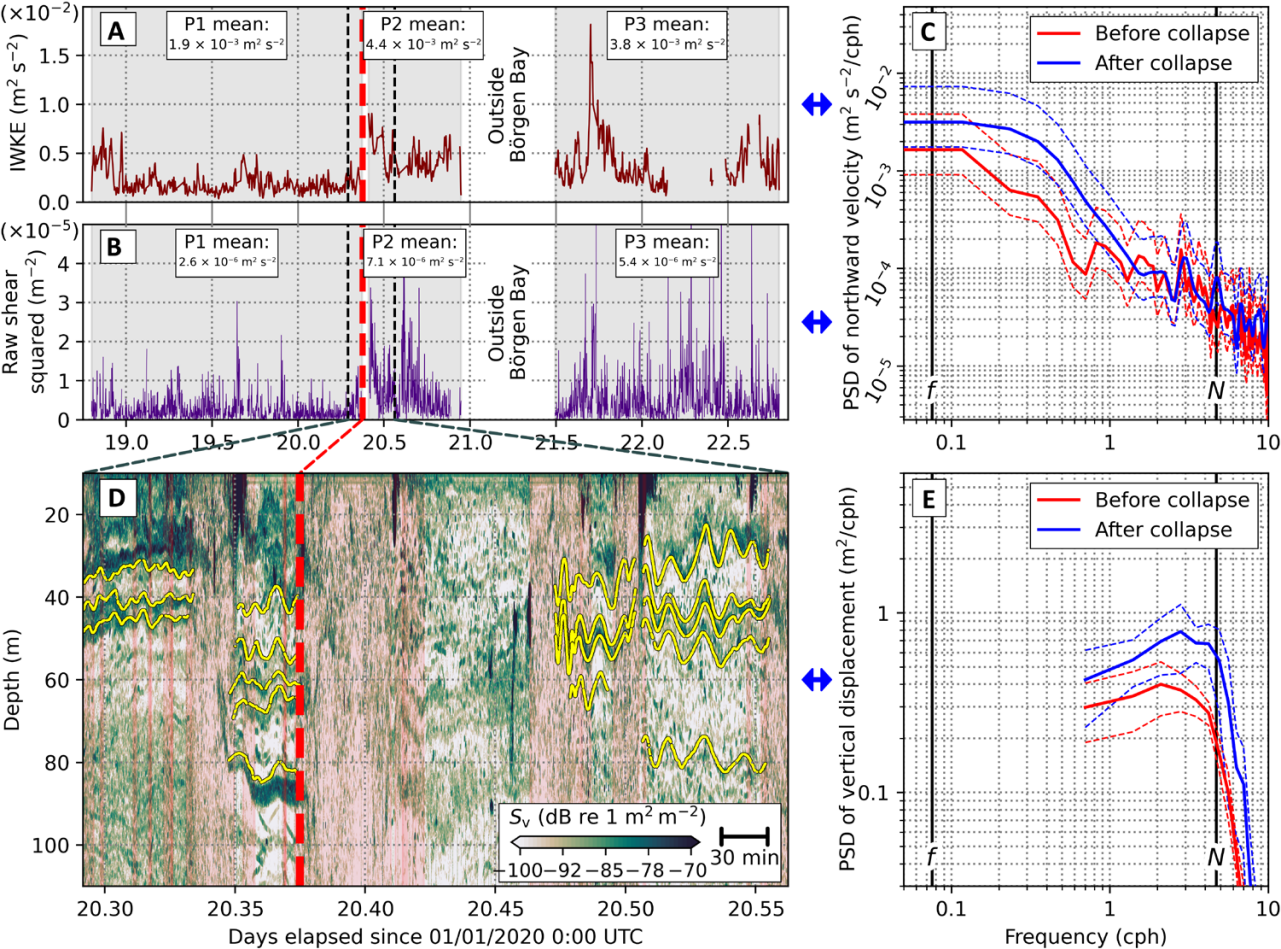
Acoustic tracking of internal tsunamis and their characteristics. (**A**) Internal wave kinetic energy (IWKE) for depths shallower than 135 m. Legend shows IWKE values averaged for three periods, specifically before the ice collapse (days 18.80 to 20.39), immediately after (days 20.40 to 20.95), and a third period after the ship had departed and then returned to Börgen Bay (days 21.41 to 22.79). (**B**) Shear-squared, calculated between 75 and 25 m depth. Note the strong increase at the time of the calving event (vertical dashed bar) and persistent elevated values thereafter. (**C**) Power spectral density (PSD) of the velocity data between 50 and 100 m depth, with uncertainty bands. (**D**) EK80 echogram for the period immediately spanning the glacier calving event. Vertical displacements of internal surfaces are traced (yellow; see Materials and Methods). (**E**) Power spectral density (PSD) of EK80-derived internal surface displacements with uncertainty bands. Frequencies <0.7 cycles per hour (cph) are not visible in the spectra because of the limited presence of clear and continuous scattering layers in the echograms.

The data gathered indicate that the observed destratification was caused by the generation, rapid propagation, and breaking of large-amplitude internal tsunami waves triggered by the calving event. Internal wave kinetic energy (IWKE; Fig. 6A) calculated from ocean velocity data (see Materials and Methods) more than doubled at the time of the calving and remained elevated for at least 2 days subsequently, with the residual stratification supporting the wave propagation. The energization of the internal wave field was further evidenced using high-frequency EK80 echosounder data (see Materials and Methods); these revealed a marked increase in the amplitude of internal layer displacements (Fig. 6D), with a corresponding increase in power at frequencies characteristic of internal waves (Fig. 6E). Upper-ocean shear squared approximately doubled at the time of the calving event, coincident with a sharp spike in IWKE (Fig. 6), indicating that the IWKE increase drove an enhancement in fine-scale shear that ultimately was the source of the turbulent mixing and destratification.

That layer displacements, IWKE, and shear were energized across the entirety of Börgen Bay is indicated by their persistently elevated values after the calving event (Fig. 6), during which time the research vessel traversed most of the fjord (c.f. fig. S3). Propagation of internal tsunamis beyond the mouth of Börgen Bay will undoubtedly have occurred, although complex topography will have led to some level of wave reflection and retention of energy within the fjord itself; without suitable measurements outside the fjord, we cannot address this directly. We note, however, that surface tsunamis and internal waves, where unobstructed, can traverse very large distances in the ocean (hundreds of kilometers or more, in extremis). Accordingly, calving in fjords that are relatively open to the shelf and that feature comparatively smooth topography could drive internal tsunamis with the potential to influence the ocean over significant distances.

**Fig. 7.**
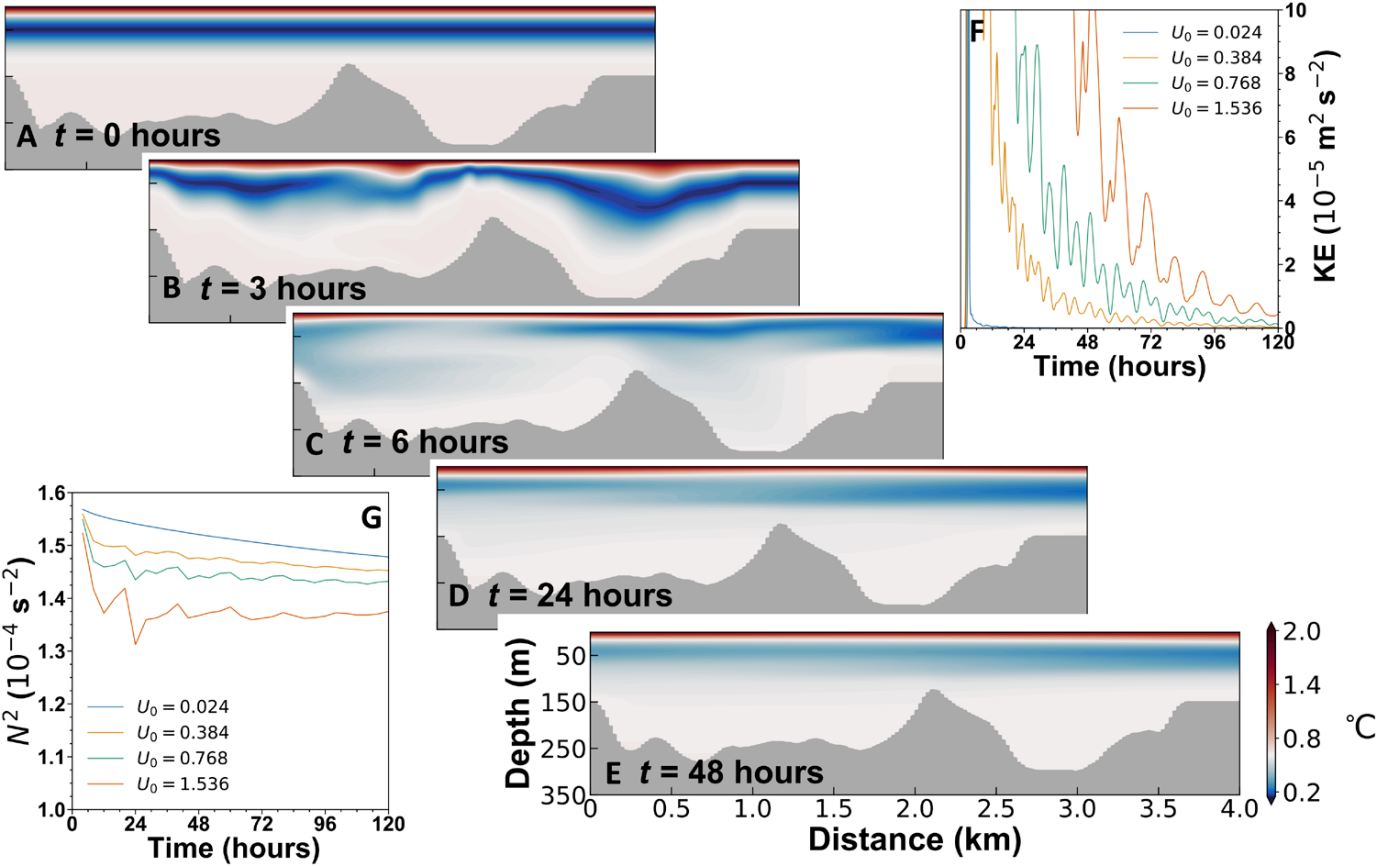
2D model simulations of internal tsunami propagation, breaking, and mixing. (**A** to **E**) Potential temperature at the time intervals stated, with internal wave introduced on the right side of the panels. The peak inflow for the imposed wave is *U*_0_ = 1.536 m s^−1^, and the total energy introduced is ~500 J m^−3^. The incoming wave peaks at 3 hours and by 6 hours are effectively zero. Note the nonlinear color scale, emphasizing where most of the mixing occurs. (**F**) Domain-average kinetic energy for four different strengths. (**G**) Four-hour averages of squared buoyancy frequency, averaged over the top 60 m.

That the high ocean energy levels and rapid mixing are consistent with the propagation and breaking of an internal tsunami is confirmed with numerical modeling (see Materials and Methods). With initial ocean properties taken from precalving observations, a very short–time scale impulsive wave is introduced at the glacier terminus of a 2D (in the meridional-vertical plane) model of Börgen Bay to represent tsunamigenesis. The model solves the hydrostatic primitive equations using finite volume methods. A nonlinear equation of state is used for density, and small-scale vertical mixing is parameterized (see Materials and Methods for full details). The internal tsunami rapidly causes isopycnal heave and mixing (Fig. 7, A and B). Subsequently, relaxation of the heave causes further mixing (Fig. 7C). Consistent with observations, during the following ~2 days, the sustained elevated energy and turbulence continue to act on the stratification, causing further homogenization (Fig. 7, D and E).

The strength of the internal tsunami is a key factor in determining the kinetic energy levels in the model and the rapidity/magnitude of destratification. For the weakest waves modeled, kinetic energy drops rapidly to near zero (Fig. 7F) and causes little mixing (Fig. 7G). Progressively stronger imposed waves give elevated levels of kinetic energy and more vigorous and rapid mixing. Model experiments with altered bathymetry reveal the importance of wave/seabed interactions in determining the structure and magnitude of mixing (figs. S5 and S6).

### Quantitative importance as a source of mixing

To determine the quantitative importance of the mixing attributable to the internal tsunamis, we compare the energy dissipation with that from other leading processes. Considered over the time interval of conductivity-temperature-depth (CTD) casts spanning the calving event (61 hours), the internal tsunami-attributed dissipation is 1.34 × 10^−1^ W m^−2^. Alternatively, using the 27.5-hour interval between the calving event and the second CTD cast yields 3.07 × 10^−1^ W m^−2^. These compare with dissipation associated with (i) pycnocline mixing due to tidally induced breaking internal waves (3.41 × 10^−5^ W m^−2^), (ii) mixing due to friction of tidal currents over the seabed (1.94 × 10^−7^ W m^−2^), (iii) wind-driven mixing at the base of the surface mixed layer (2.25 × 10^−4^ W m^−2^), and (iv) annual surface cooling–induced mixing (5.07 × 10^−4^ W m^−2^) (see Materials and Methods for derivation of these terms and confidence intervals; Table 1). The latter term can be divided into summer and winter components of 2.16 × 10^−4^ and 7.44 × 10^−4^, respectively (Table 1). It is clear that, during the summer period when internal tsunami-induced mixing is most active, it dominates these other processes by at least two to three orders of magnitude. A key difference between these sources of mixing is that glacier calving is episodic, unlike winds and tides, which are (quasi-)continuous. Nonetheless, taken over a summer season (when stratification is strongest and internal tsunamis can have most effect), just one calving event of the scale witnessed would result in internal tsunamigenesis being at least equivalent to winds and buoyancy forcing in driving mixing and much more important than tides. Typical calving frequencies comfortably achieve or exceed this (see Materials and Methods; Fig. 2).

**Table 1.** Terms for comparison with dissipation attributed to the internal tsunami, namely (from top) dissipation due to breaking internal tides, dissipation due to bed friction, dissipation due to wind-driven mixing, and dissipation due to cooling-induced mixing. The latter term includes annual, winter, and summer quantifications. CI, confidence interval.

	**Mean dissipation (Wm^−2^)**	**95% CI (lower bound)**	**95% CI (upper bound)**
*P*_IW_	3.41 × 10^−5^	3.24 × 10^−5^	3.57 × 10^−5^
*P*_BBL_	1.94 × 10^−7^	1.88 × 10^−7^	2.00 × 10^−7^
*P*_W_	2.25 × 10^−4^	2.04 × 10^−4^	2.50 × 10^−4^
*P*_B_ annual	5.07 × 10^−4^	4.88 × 10^−4^	5.26 × 10^−4^
*P*_B_ winter	7.44 × 10^−4^	7.17 × 10^−4^	7.72 × 10^−4^
*P*_B_ summer	2.16 × 10^−4^	2.00 × 10^−4^	2.31 × 10^−4^

## DISCUSSION

Internal tsunamis are likely widespread wherever marine-terminating glaciers actively calve, including large regions of Antarctica, Patagonia, Alaska, Canada, Iceland, Russia, Svalbard, and Greenland. The generation of surface tsunamis by calving events across these locations is known (*20*–*22*); the absence of comparative literature on internal tsunamis does not, we argue, reflect their sparsity but instead a paucity of appropriate observations.

The full coastline of Antarctica (43,449 km; (*23*)) includes approximately 75% floating ice shelves, with the remainder being ice walls, a few thousand other glaciers, and <1% rock. In steady state, the ~2300 to 2500 Gt (gigatonne = 10^12^ kg) annual surface mass balance of Antarctica (*24*) is balanced by ice discharge at the periphery; very roughly half of this ice is calved into icebergs (*4*, *25*, *26*). Elsewhere, between 30 and 60% of net mass loss from the Greenland Ice Sheet are estimated to be from iceberg calving (*27*), while calving dominates glacial retreat in Patagonia (*28*). With more than 85% of the >1700 Arctic marine-terminating glaciers in retreat since 2000, calving events have been a major factor in the total area loss of 7527 ± 31 km^2^ (*29*).

The dimensions of icebergs calved from Antarctica follow a well-established power law, with larger icebergs being more massive but calving less frequently than smaller ones (*30*); however, it is not the case that tsunamigenesis and ocean mixing will also scale accordingly. Calving of large tabular icebergs from ice shelves likely has a very different impact on ocean mixing than does grounded marine-terminating glaciers: The ice is already afloat, and the injection of potential energy to the ocean will not happen instantaneously or necessarily locally. Nonetheless, calving events of the approximate scale of our Börgen Bay event are frequent, with possibly thousands per year [estimates for the number of glaciers around Antarctica range from 2752 (*31*) to 3274 (*32*), with the latter figure including the subantarctic region]. We expect the Antarctic Peninsula to be a hot spot of glacier calving on this scale, with marine-terminating glaciers widespread and the large majority in retreat (*19*). Accordingly, it is highly likely that internal tsunami events of the scale witnessed are frequent and widespread.

The injection of strong mixing energy at the ocean/ice interface and across the shelf has significant implications, including for the supply of nutrients to the euphotic zone and for upper-ocean stability and hence light availability for phytoplankton growth. Both processes affect primary production and consequent carbon drawdown. We examined this in our biogeochemical data, but the short duration of our measurements after the calving event (~2 days) precluded definitive results. In addition, the redistribution of heat in the ocean will affect surface temperatures, sea ice production, the pattern of glacier front melt, and water mass transformations. Given the widespread nature and frequency of calving events of the form observed, the collective impact of the internal tsunamis created could have marked consequences for the cryosphere, carbon drawdown, and climate.

There is the potential for climatic modulations to internal tsunamigenesis and mixing, because glacier calving can be sensitive to both ocean and atmospheric temperatures (*2*, *25*, *33*–*35*). This is exemplified by the West Antarctic Peninsula, which has warmed significantly since the middle of the last century (*36*, *37*), with glacier retreats accelerating (*19*). This suggests the possibility of long-term changes in internal tsunamigenesis and mixing, both at the Peninsula and elsewhere as decadal warming progresses, although nonuniformity in calving response and the potential for changing glacier geometry challenge simple projections/extrapolations. For glaciers that retreat sufficiently to become land terminating, this source of mixing will eventually be nullified. Improved knowledge of the future of internal tsunamigenesis and mixing is important; however, the current generation of ocean/climate models do not include these processes; developing their capability is a priority.

## MATERIALS AND METHODS

### Satellite imagery and calculation of energy from calving

Satellite imagery from Landsat 8 reveals the spatial scale of the William Glacier calving event (Fig. 1C), with images collected precalving on 17 January 2020 and postcalving on 24 January 2020. These images were obtained courtesy of the U.S. Geological Survey. Comparison of the images reveals that the surface area of glacier loss upon calving was approximately 78,000 m^2^. The average height of the glacier above sea level was ~42 m, as derived from the Reference Elevation Model of Antarctica (*38*) and converting from the WGS84 ellipsoid to height above mean sea level using the EGM2008 model. Accordingly, the volume of ice discharged from above the sea surface can be approximated as 3.3 × 10^6^ m^3^, equivalent to a mass of around 3 × 10^9^ kg if mean ice density is ~900 kg m^−3^. Converting this mass to gravitational energy release upon calving yields a value of approximately 6 × 10^11^ J.

There is more uncertainty concerning the volume of ice calved from below sea level, because the glacier here is not directly visible in satellite imagery. If it is presumed that the glacier front was vertical and that all the ice below sea level was released upon calving, the volume of ice released can be calculated as 1.7 × 10^7^ m^3^ based on a mean glacier depth at the calving front of 210 m as measured with ship multibeam echosounder data. Using Archimedes’ principle, by which the upward buoyant force depends on the difference in density between ice and the water that it has displaced, the buoyant force can be estimated as ~1.8 × 10^10^ N. Over the mean distance of uplift, an upper limit on the energy released from calving below sea level can thus be estimated at 1.8 × 10^12^ J.

While these estimates are necessarily coarse, they do provide order of magnitude information on the amount of energy released from the calving, namely, (0.6 to 2.4) × 10^12^ J. Our presumption is that the actual figure for energy release will be close to the upper limit of this range, because it seems likely that most of the available ice from below sea level was calved. From the time-lapse analysis of surface crevasse displacement (fig. S1), ice flow increases toward the terminus in Börgen Bay. These longitudinal velocity gradients enable crevasses to reach deep into the glacier while the ice is still grounded, favoring full-thickness calving. Where crevasses are less prominent, the shape of the profile of the grounded ice front (fig. S1) suggests that extensive melt undercutting is unlikely the driving mechanism of calving [c.f. *39*], and, if there is buoyant calving, that half of the ice thickness would still be involved (fig. S1).

For comparison with ocean potential energy and internal wave energy derived from shipboard measurements, if this calving energy were spread equally over the surface area of Börgen Bay (34.8 km^2^) and the upper 180 m of the water column, then it would equate to an approximate energy density of 90 to 380 J m^−3^. The fate of this energy will include sound, heat through friction, surface waves, internal waves, and turbulent mixing. Our contention is that the latter two terms are significant.

### Acquisition and processing of cruise data

Ocean profile and underway data were collected on cruise JR19002 of the research vessel RRS *James Clark Ross*, 31 January 2019 to 4 February 2020. Data used here were collected within Börgen Bay (West Antarctic Peninsula) during 19 January to 24 January 2020.

Vertical profile data were collected with a SeaBird 911+ CTD instrument, mounted on a 24-bottle rosette frame fitted with 10-liter Niskin bottles. Direct sampling from the Niskins included salinity samples, which were analyzed on an Autosal 8400B for calibration/checking of the CTD data. Separately, a SeaBird SBE35 high-precision thermometer was used for calibration/checking of the CTD temperature data. Other instruments mounted on the frame included a C-Star transmissometer.

Underway data collected included thermosalinograph data from a SeaBird SBE45 CTD system and Vessel-Mounted Acoustic Doppler Current Profiler (VM-ADCP) data collected with an RDI 75-kHz Ocean Surveyor. Thermosalinograph salinity was calibrated with discrete salinity samples; VM-ADCP data were processed and calibrated using the CODAS processing software suite (*40*).

To calculate the IWKE, the processed 2-min 75-kHz ADCP data were first gap-filled using a linear interpolation in the top 135 m (at depths greater than 135 m, there were insufficient high-quality data to do this successfully). The zonal and meridional (*u* and *v*) velocities were then band-pass–filtered between the inertial frequency (*f* = 1.3 × 10^−4^ s^−1^) and the buoyancy frequency (*N*) using a symmetric fourth-order Butterworth filter. For times when *N* was higher than the Nyquist frequency, a simple high-pass Butterworth filter was instead used. IWKE was then calculated as the depth average from the surface to 135 m of 0.5 (*u*^2^ + *v*^2^). The mean IWKE was calculated for each period as outlined in the main text. To test the sensitivity of the calculation to whether the ship was moving or not, we computed the means using all the data and only those periods where the ship speed was less than 0.1 ms^−1^. No significant difference was observed using the two different criteria.

To calculate shear squared between 25 and 75 m, *u* and *v* shears were calculated using depth-averaged 20- to 30-m and 70- to 80-m velocities before being summed and squared. After detrending the gap-filled *u* and *v*, power spectra were calculated using Welch’s method (eight 50% overlapping Hamming windows) for each of the three periods outlined in the main text.

High-frequency echo sounder data were acquired with a Simrad EK80 scientific echo sounder running near continuously, including during the ice front collapse at Börgen Bay. Three transducers were active at 38, 70, and 120 kHz, respectively. Parameters from a calibration near the end of the survey, using a sphere with a known target strength, were applied in postprocessing. Interfering EA600 echosounder pings were removed in Echoview software based on the impulsive noise algorithm by Ryan *et al.* (*41*) and a further median filter. Background noise was attenuated on the basis of the method of de Robertis and Higginbottom (*42*).

For internal wave identification in the EK80 data, we disregarded the ratio of contribution of biological and physical sources of the backscattering strength or the taxonomic composition and treated the vertical displacement of the scattering layers as directly linked to vertical displacement of physical stratification. Although biological targets are not immobile, we assume that the averaged movement of mesozooplankton, macrozooplankton, and micronekton, which are generally imaged in sound scattering layers [e.g., *43*, *44*], does not significantly affect the resulting vertical displacement time series at frequencies associated with the internal wave regime. The depth of reflectors showing internal wave features was picked manually as segments of time series and detrended using a least squares linear regression. Data were filtered with a low-pass Butterworth filter of 20 cycles/hour to eliminate signals associated with picking inaccuracies and noise. Power spectral densities were computed using the multitaper method (*45*, *46*) with four Slepian tapers.

Clear and continuous scattering layers are necessary for analysis of the durations of picked vertical displacement time series in the echogram; because these are limited, frequencies of <0.7 cycles/hour are not visible in the frequency spectrum and a full quantification of internal wave energy is not possible with the EK80 data. Furthermore, an increase in more chaotic scattering throughout the water column was observed during a stationary phase following the ice front collapse, potentially a result of breaking internal waves.

The Kongsberg EM122 multibeam echo sounder, hull-mounted on RRS *James Clark Ross*, provided depth values to both the seabed and the grounded ice front. Using the QPS Qimera software, a weak spline filter was applied to discard spurious data, and all point data rejections were checked manually with ice front soundings being reinstated. Sound velocity profiles were obtained to accurately convert measured time to depth; these were generated via CTD profiles of conductivity, temperature, and depth. Depth values were corrected for variations in tidal height using real-time tidal observations from the nearby Palmer Station. The final seabed bathymetry raster has a cell size of 5 m, while the shape of the grounded ice front (from ~50 m in depth) was investigated from the point cloud directly.

### Quantifying changes in ocean potential energy anomaly

CTD casts before (Station BBA) and after (Station BBB) the calving event were used to quantify changes in ocean potential energy anomaly (Δϕ). These stations were both within the fjord but separated by ~400 m, with the time interval between their occupation being 61 hours, and with the second CTD cast taken 27.5 hours after the calving event. To mitigate against single-cast aliasing of internal wave isopycnal heave, cast BBA was advected vertically with the mode 1 vertical velocity profile such that mass was conserved before and after calving, as per Inall *et al.* (*47*). To account for the spatial separation of the casts in a fjord with a nonzero horizontal salinity gradient (noting that salinity controls density), BBB salinity was first adjusted with a constant offset of 0.059. Without this adjustment, the mass conservation vertical heave compensation would unphysically transpose a known horizontal density gradient into exaggerated vertical heave. The maximum vertical displacement of heave adjustment was −18.3 m (i.e., downward) centered at ~80 m depth, a value consistent with the magnitude of vertical displacements observed in EK80 echosounder data. Last, to calculate the turbulent kinetic energy made available by the internal tsunami (i.e., the tsunami-attributed dissipation), we assume that 20% is converted to changing the potential energy of the system (i.e., Δϕ) and 80% dissipated as heat (*48*). Internal tsunami-attributed dissipation values are converted to units of watt per square meter for comparison with other dissipation processes using CTD and calving event times.

With few postcalving CTD profiles, the level to which they represent the fjord-wide change in properties is hard to quantify absolutely. However, it should be recognized that there is nothing anomalous about the CTD station locations, and the underway data (fig. S3), while near-surface only, indicate a high level of fjord-wide uniformity of the response to the calving. Second, the 2D model results indicate stratification change fully across the domain, again indicating a likely high level of representativeness of the CTDs in depicting that change.

### Numerical modeling of mixing driven by calving events

The model is a 2D slice along the central axis of Börgen Bay using the MIT general circulation model (*49*, *50*). The horizontal grid spacing is 23.7 m over the central 180 grid boxes of the domain. It increases linearly to 237 m over the next 20 grid boxes and maintains this size for the last 10 grid boxes. The increase in horizontal grid spacing takes place at both ends of the domain, for a total of 240 grid boxes. This is intended to help reduce reflection from either end of the model, although some degree of reflection would be expected in the fully 3D setting (in the complex environment of Börgen Bay, convoluted bathymetry, coastlines, and the presence of external islands would lead to some reflection of energy within and back into the bay). The vertical grid spacing is 2 m with a maximum allowed depth of 340 m, and the model is run in hydrostatic mode. The bathymetry is smoothed with a three-point box filter to prevent numerical instability. Each model experiment is run for 5 days with a timestep of 0.125 s.

The model uses the nonlinear equation of state of Jackett and McDougall (*51*) with the actual hydrostatic pressure applied at each timestep. The initial temperature and salinity profiles are idealized versions of CTD profiles from station BBX. They are designed to remove small-scale static instability that would otherwise generate motion while closely matching surface values and the positions of local maxima deeper in the water column.

To prevent spurious mixing due to heaving of isopycnals across model levels, we use the r* vertical coordinate system (*52*) with partial cells (*53*). With *z* level coordinates, this spurious mixing is found to be particularly egregious near the surface, such that it dominates the total mixing in the channel regardless of the strength of the input wave (see below). To further reduce any spurious mixing due to small-scale noise in the temperature and salinity, we use the seventh-order tracer advection scheme due to Daru and Tenaud (*54*). In larger-scale problems, this scheme is found to help ensure a quasi-adiabatic circulation (*55*). The horizontal viscosity and diffusivity, on both temperature and salinity, are set to the small value of 5 m^2^ s^−1^. Similarly, the vertical viscosity and diffusivity for both temperature and salinity are set to 1 × 10^−5^ m^2^ s^−1^. Additional vertical mixing is provided by the parameterization of Pacanowski and Philander (*56*).

To generate an internal tsunami in the model, a depth-independent flow is introduced at the northern end of the domain. This inflow is given by

$$U=-\frac{U_{0}}{\text{cos}\;h^{2}(\pi (t-3))}$$ (1)

where *t* is the time in hours. The peak inflow is 3 hours after model initialization, and the time scale of the decay is 2 hours; this ensures a rapid delivery of energy/momentum to the model domain. The strength of the wave is characterized by *U*_0_, with stronger waves having larger values. We perform nine experiments starting with *U*_0_ = 0.024 ms^−1^ and doubling *U*_0_ between each experiment to a maximum value of 6.144 ms^−1^. To prevent large increases in domain volume, the same flow is also prescribed at the southern end; this outflow is also depth independent.

### Comparison with noncalving processes

#### Dissipation due to breaking internal tidal waves

In shelf seas, barotropic tides lose a portion of their energy to internal tides as stratified water oscillates over bathymetric slopes, termed tidal conversion. This can be estimated from a combined knowledge of barotropic tidal currents, water column stratification, and bathymetric charts. Adopting the method of Egbert and Ray (*57*), as implemented by Green and Nycander (*58*) and adapted for shelf seas by Inall *et al.* (*59*), tidal conversion is estimated for the entire Antarctic shelf using the CATS08 inverse tidal model (*60*), GEBCO 30–arc-sec bathymetry (*61*), and two sources of water column stratification: in situ observations from Börgen Bay and the Southern Ocean State Estimate (SOSE) (*62*). First, a form drag term is computed

$$C_{\text{ZE}}=\beta H{\left(\nabla H\right)}^{2}\frac{N_{b}\bar{N}}{8\pi^{2}\omega }$$ (2)

where β is a scaling factor used to compensate for unresolved topography, *H* is the total water depth, and ω is the tidal frequency. Stratification terms are formed by fitting a horizontally homogeneous stratification *N*(*z*) = *N*_0_ exp (*z*/L_N_), where *L_N_* is the vertical decay scale and *N*_0_ is a background reference stratification. *N*_b_ = *N*(*H*), and $\bar{N}$ is the vertical average of *N*(*z*). Tidal energy conversion is then computed using the form drag in units of watts per square meter as

$$P_{\text{IW}}=\rho_{0}C_{\text{ZE}}u^{2}$$ (3)

where *u* is the barotropic tidal current speed (*60*). Four tidal constituents (M_2_ S_2_ K_1_ O_1_) are computed separately and summed to give the total conversion. The histogram of Eq. 3 for the West Antarctic Peninsula sector (60°W to 130°W) is shown in fig. S7A. A 1000-sample bootstrapping method is used to evaluate the mean and 95% confidence interval for this term and comparator terms below.

#### Mixing due to bed friction

Boundary-generated turbulence is inefficient at eroding stratification (*63*). Pycnocline mixing due to bed friction is estimated following Simpson and Bowers (*63*) as *P*_MBL_ = 0.4 × 10^−3^*P*_BBL_, where *P*_BBL_ is the rate at which energy is dissipated via boundary layer drag, estimated with units of watts per square meter following Simpson *et al.* (*64*) as

$$P_{\text{BBL}}=\rho_{w}C_{D}u^{3}$$ (4)

where ρ_w_ is water density (1025 kg m^−3^) and drag coefficient *C*_D_ = 2.5 × 10^−3^. *P*_MBL_ is estimated for the entire Antarctic shelf using the CATS08 inverse tidal model (*60*) and plotted as a histogram for the West Antarctic Peninsula shelf in fig. S7B.

#### Wind-driven mixing

From the viewpoint of wind at the reference meteorological height of 10 m above the surface, most wind kinetic energy is dissipated to heat through turbulent motions in the atmospheric and oceanic boundary layers and is therefore not available to alter water column stratification. The proportion of wind energy that is available to change ocean stratification is dependent on many factors, especially processes in the oceanic boundary layer. Following Alford (*65*), an estimation of wind-driven mixing is made here using the 1D Price-Weller-Pinkel (PWP) (*66*) ocean mixed-layer model forced by hourly ERA5 10-m winds (*67*) and initialized with both in situ observations and SOSE (*62*) temperature and salinity profiles. To isolate water column mixing due only to wind work, all surface buoyancy fluxes are set to zero, the PWP model run for 30 days, and the time rate of change of the PE anomaly (units watts per square meter) computed hourly, with wind mixing given by Alford (*65*)

$$P_{W}=\frac{d}{dt}\text{PE}$$ (5)

The histogram of Eq. 5 for the West Antarctic Peninsula sector is shown in fig. S7C.

#### Buoyancy forcing

We have estimated the impact of buoyancy forcing (*P*_B_) for calendar year 2020 by applying equation 8 of Alford (*65*) and using hourly values for mixed layer depth predicted by the PWP model forced with ERA5 buoyancy and momentum fluxes (*67*). This gives an estimate of the rate of increase of water column potential energy due to convective entrainment of deeper waters into the surface mixed layer driven by surface buoyancy loss due to net cooling. Values are given in watts per square meter, directly comparable with other mixing rate estimates here. We exclude all times when surface buoyancy flux is negative (net heating), and we further separate estimates into summer (September to February) and winter (March to August). Histograms of this term (both annual and seasonal values) are given in fig. S7D.

#### Relative importance of terms

The four mixing terms evaluated above (in units of dissipation, watts per square meter) are given in Table 1. All represent area averages over the West Antarctic Peninsula shelf. By two and three orders of magnitude, respectively, wind-mixing and annual buoyancy forcing dominate over internal tide mixing and bed friction mixing. In summer, when calving is most frequent, convective mixing and wind mixing are similar. In winter, when calving is least frequent, convective mixing dominates by a factor of ~3.

The equivalent dissipation due to breaking of the internal tsunami, averaged over the 61-hour interval between bracketing CTD casts, is 1.34 × 10^−1^ Wm^−2^. Alternatively, using the interval between the calving event and the second CTD cast (27.5 hours) yields 3.07 × 10^−1^ Wm^−2^.

Some other mixing processes should be mentioned but are not quantified here. Dense water overflows between topographically isolated basins are capable of modifying stratification locally through entrainment and advection; their effects on Antarctic shelves have been documented (*68*) but remain unquantified. In Greenlandic fjords, the inverse process of subglacial freshwater discharge exerts an influence on fjord water stratification and subsequent circulation (*69*, *70*) but is not considered here.

### Calving frequency of the William Glacier

Glacier terminus position is measured at high temporal resolution using a time series of Sentinel-1 synthetic aperture radar (SAR) images acquired between 3 May 2015 and 28 December 2021. These data provide year-round, cloud-free radar images of the glacier terminus and Börgen Bay with revisit intervals of between 12 and 1 day(s). The glacier terminus is digitized in each image using the Sentinel-1 Ground Range Detected data product in Google Earth Engine using the GEEDiT digitization tool (*71*). From the March 2021 images onward, this tool could not be used due to a georeferencing error in Google Earth Engine and digitization was performed locally at 12-day intervals. From these digitizations, terminus change is then calculated relative to the first image using the “curvilinear” box method (*71*), which is an extension of the “box” method (*72*). This averages terminus change over a 2000-m-wide box covering the glacier front; this provides a more robust measurement than a centerline method. Errors in manual digitization are approximately equal to image pixel size (±10 m) (*73*).

## Supplementary Materials

### This PDF file includes:

Figs. S1 to S7 References
