# Supplementary material for: Internal tsunamigenesis and ocean mixing driven by glacier calving in Antarctica
Source: Sci Adv. 2022 Nov 23;8(47):eadd0720. doi: 10.1126/sciadv.add0720 (PMC9683708; doi:10.1126/sciadv.add0720)
Supplement: Supplementary file 1 — Figs. S1 to S7 References [file sciadv.add0720_sm.pdf]

Science Advances
AAAS

Supplementary Materials for

Internal tsunamigenesis and ocean mixing driven by glacier calving in Antarctica

Michael P. Meredith *et al.*

Corresponding author: Michael P. Meredith, mmm@bas.ac.uk

Sci. Adv. **8**, eadd0720 (2022)
DOI: 10.1126/sciadv.add0720

This PDF file includes:

Figs. S1 to S7
References

Supplementary Materials

Supplementary Figures

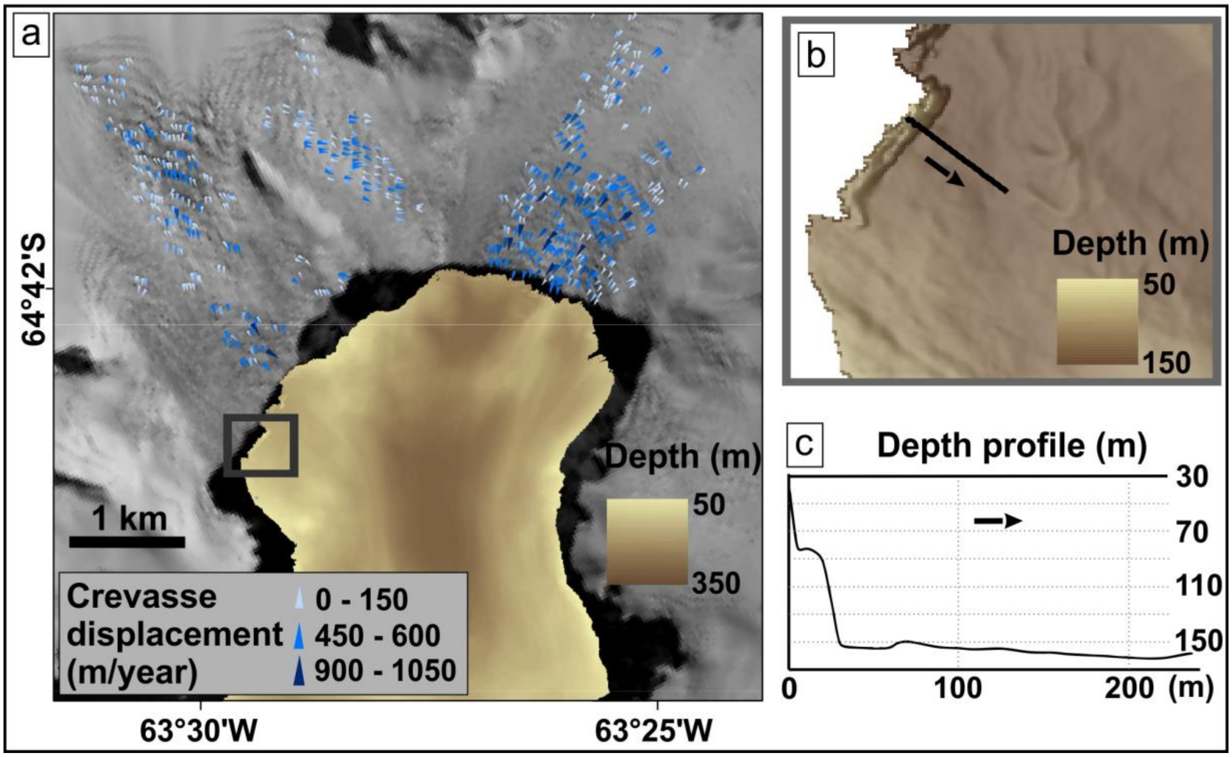

Fig. S1. Bathymetry and crevasse displacement rate for William Glacier. (a) Seabed bathymetry of Börgen Bay from multibeam echosounder data, and coastline/topography from Landsat imagery, with crevasse displacement rate and location shown in panel b. (b) Location of the depth profile of the grounded ice front presented in panel c. (c) Depth profile of the grounded ice front.

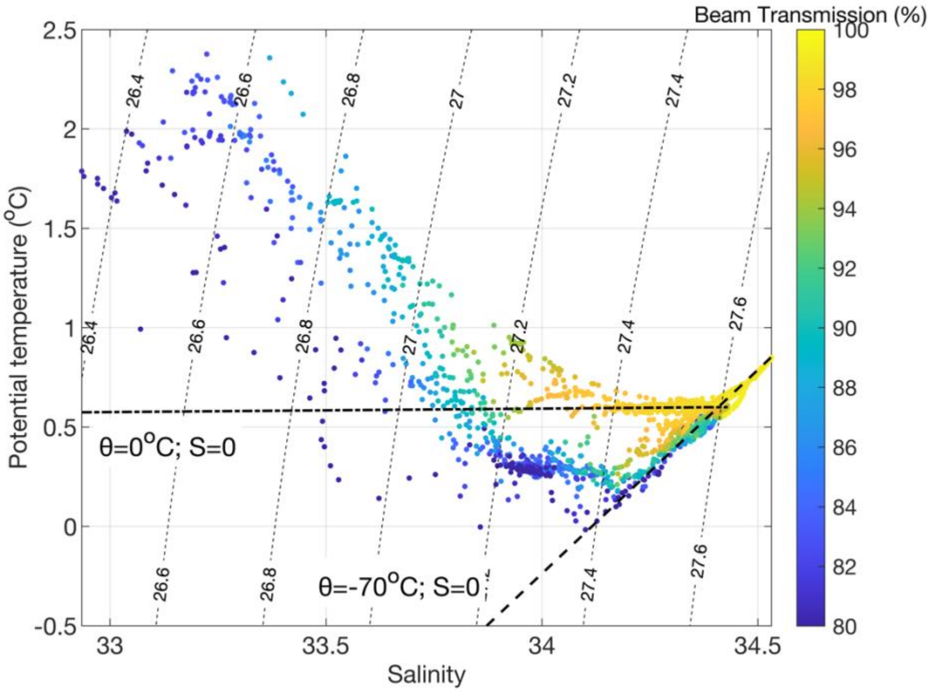

Fig. S2. Börgen Bay oceanographic characteristics. Potential temperature versus salinity for the Börgen Bay CTD profiles, coloured by beam transmission. Contours of potential density anomaly are marked. High beam transmission denotes clearer waters; low beam transmission in deeper (denser) waters denotes waters with comparatively high concentration of sedimentary particles. Glacial meltwater lies along the steep diagonal dashed line defined by Gade (*75*), whilst subglacial discharge lies along the near-horizontal dashed line. The data points with low beam transmission lie along the Gade (*75*) line at depth; these are stations featuring strong glacial meltwater input, which has low beam transmission due to the presence of sedimentary particles in the water. This water rises at the front of William Glacier and spreads as a plume across Börgen Bay at approximately 50m depth. The mixing caused by the glacier calving resulted in the locus of points lying above both lines.

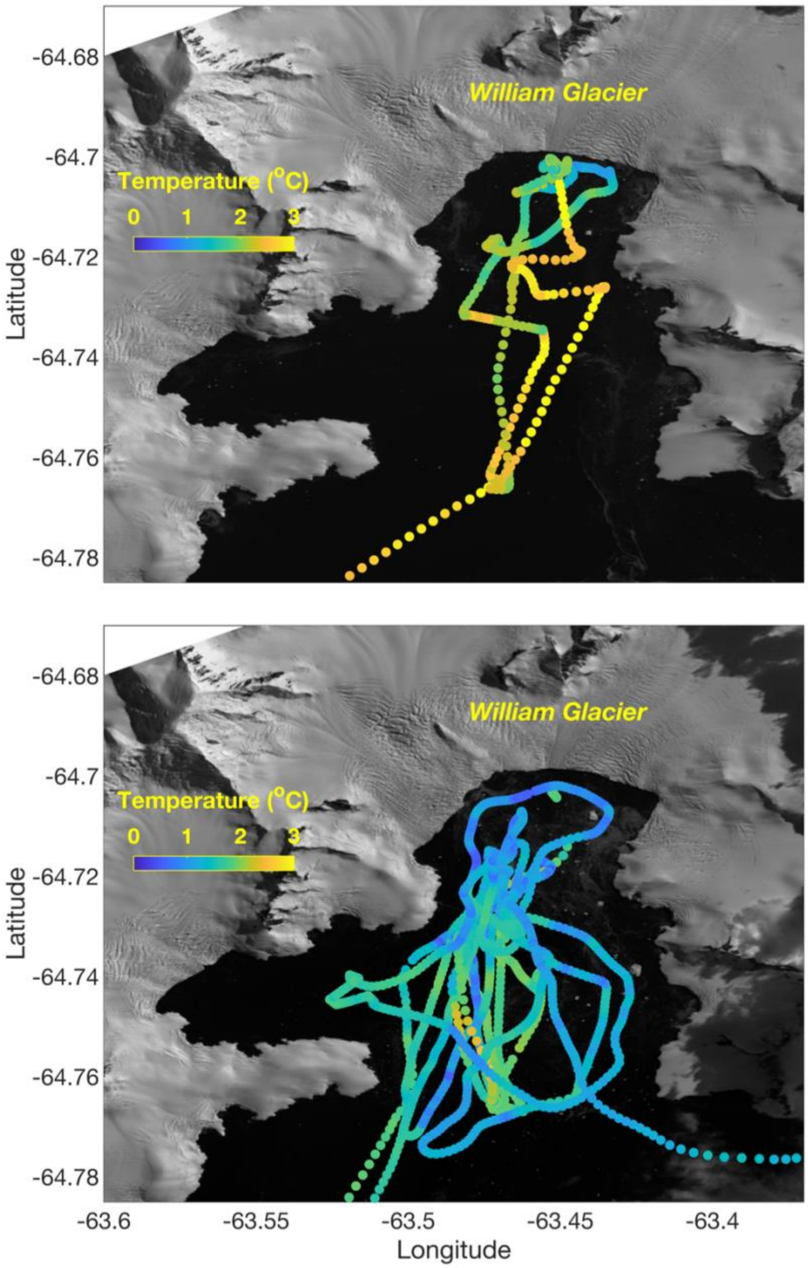

Fig. S3. Near-surface underway hydrographic data. Underway near-surface temperature along the ship track in Börgen Bay, for the periods before (upper panel) and after (lower panel) the calving event. The marked decrease in temperature at the time of the calving event is apparent, consistent with upward mixing of cooler waters from below.

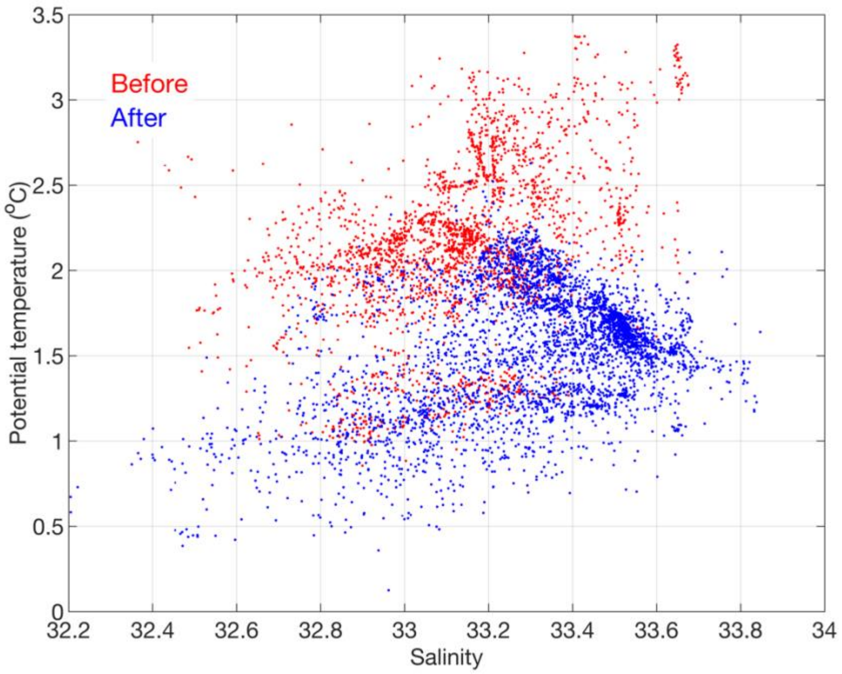

Fig. S4. **Near-surface underway hydrographic data in temperature-salinity space**. The change in upper-layer temperature (Fig. S3) is concurrent with a shift toward higher salinity, indicating that it was caused by upwards mixing of more saline water below, as opposed to injection of cold (fresh) meltwater at the surface.

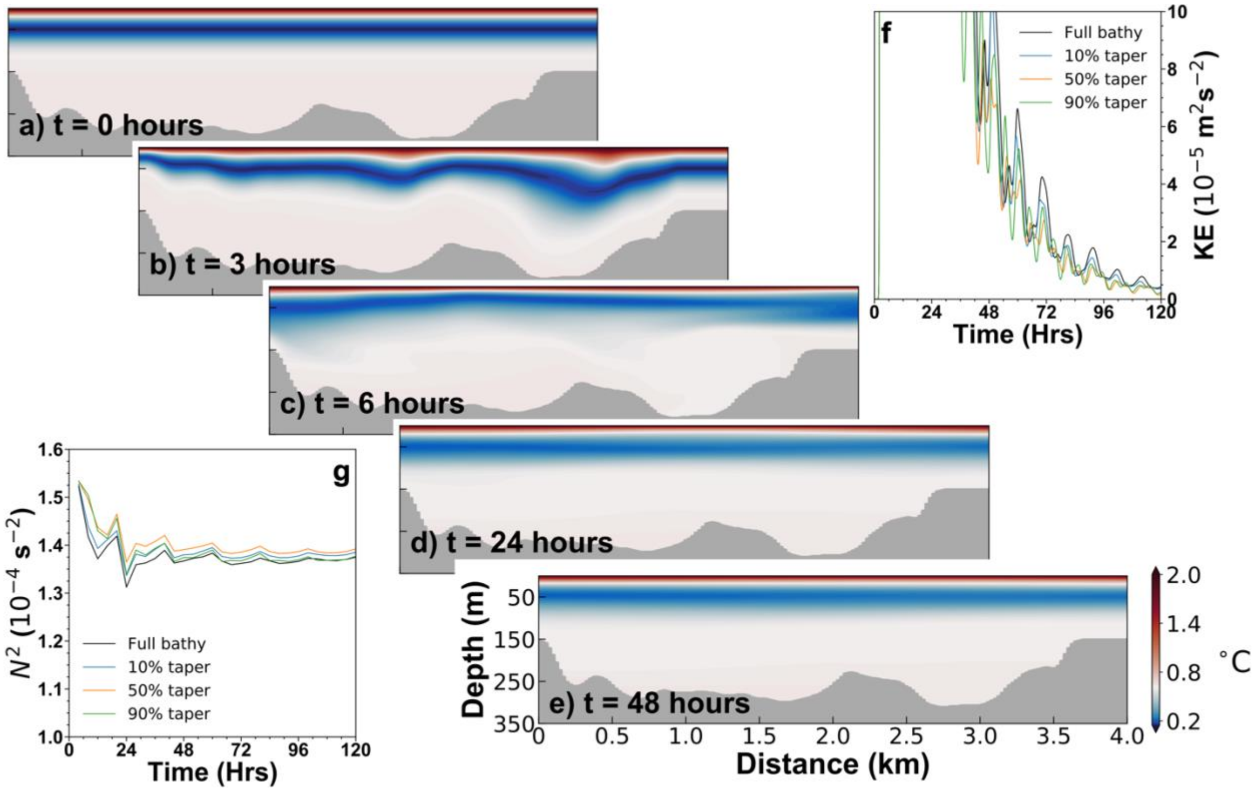

Fig. S5: Numerical simulations with 50% tapered bathymetry. As for Fig. 7, but with the bathymetry in the centre of the domain tapered by 50% towards a flat bottom. (a-e) Potential temperature at the time intervals given in the individual panels. The peak inflow for the imposed wave is U_0 = 1.536 m s^{-1} and the central ridge has been tapered to 50% of its height from the bottom. At 3 hours the impulsive flow reaches its peak velocity and by 6 hours it is effectively zero. Note the nonlinear colour scale, which emphasises the temperatures where most of the mixing takes place. (f) Domain average kinetic energy for 4 different tapers as per the legend. (g) A series of 4-hour averages of squared buoyancy frequency, spatially averaged over the top 60 m.

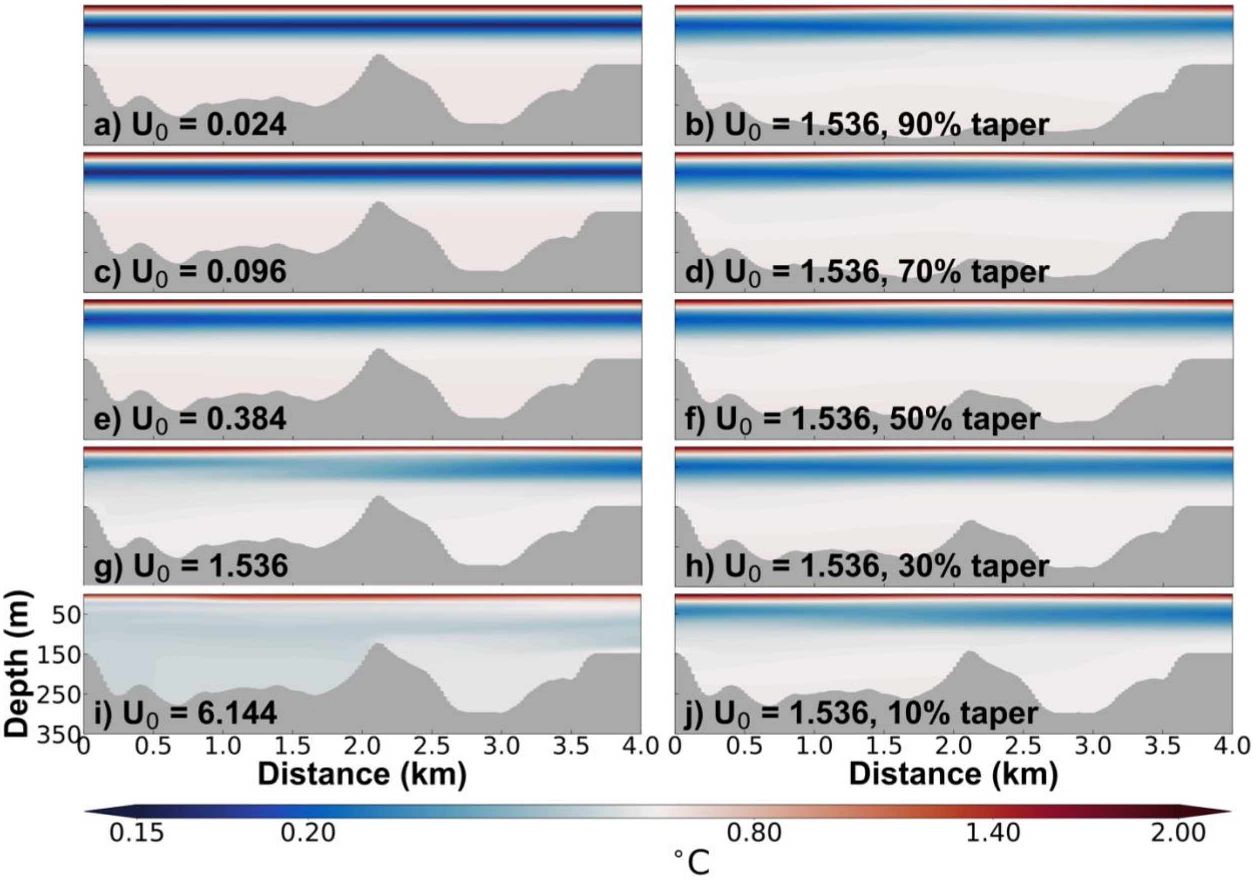

Fig. S6: Numerical simulations with varying wave strengths and bathymetric tapers. 2D model potential temperature after 24 hours for a range of model parameters. (a,c,e,g,i) varies the strength of the incoming wave, as per the individual captions; mixing between 50 and 100 m is seen to be critically dependent upon this strength. For (b,d,f,h,j), the strength of the wave is held constant at 1.536 m s^{-1} and the height of the central ridge is tapered towards a flat bottom by the percentage in the individual caption. Note the progressively weaker effect of the mixing as the bathymetric taper is increased.

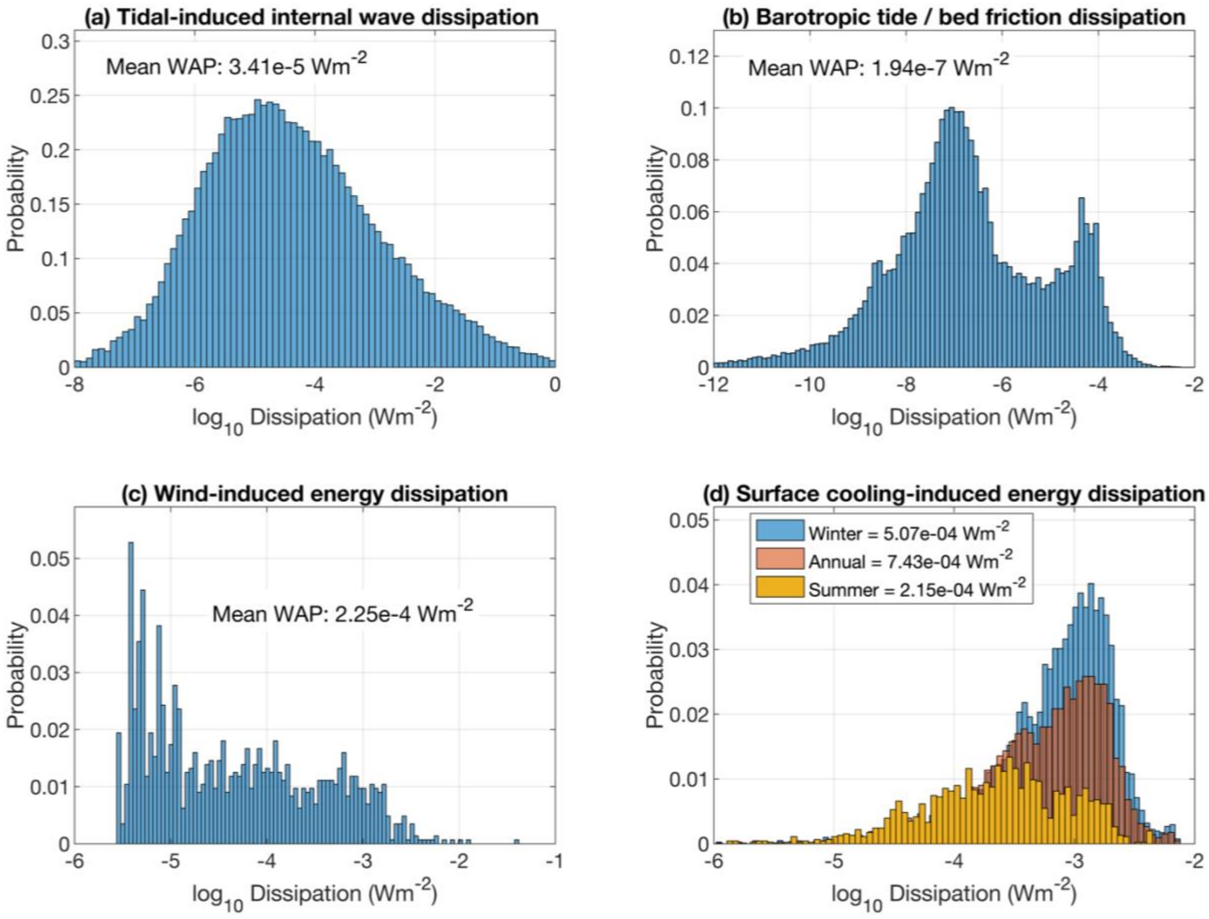

Fig. S7. Histograms of dissipation due to non-tsunami processes. (a) Histogram of tidal conversion computed at 30 arc-second resolution. (b) Bed friction dissipation. (c) Wind / near-inertial shear-induced dissipation. (d) Surface cooling-induced dissipation. In each panel, mean values for the West Antarctic Peninsula shelf are stated.

REFERENCES AND NOTES

1. M. P. Meredith, M. Sommerkorn, S. Cassotta, C. Derksen, A. Ekaykin, A. Hollowed, G. Kofinas, A. Mackintosh, J. Melbourne-Thomas, M. M. C. Muelbert, G. Ottersen, H. Pritchard, E. A. G. Schuur, Polar Regions, in *IPCC Special Report on the Ocean and Cryosphere in a Changing Climate*, H-O. Portner, D.C.Roberts, V. Masson-Delmotte, P. Zhai, M. Tignor, E. Poloczanska, K. Mintenbeck, A. Alegria, M. Nicolai, A. Okem, J. Petzold, B. Rama, N.M. Weyer, Eds. (Cambridge Univ. Press, 2019), pp. 203–320.

2. A. J. Cook, P. R. Holland, M. P. Meredith, T. Murray, A. Luckman, D. G. Vaughan, Ocean forcing of glacier retreat in the western Antarctic Peninsula. *Science* **353**, 283–286 (2016).

3. A. Shepherd, D. Wingham, E. Rignot, Warm ocean is eroding West Antarctic Ice Sheet. *Geophys. Res. Lett.* **31**, L23402 (2004).

4. E. Rignot, S. Jacobs, J. Mouginot, B. Scheuchl, Ice-Shelf Melting Around Antarctica. *Science* **341**, 266–270 (2013).

5. The IMBIE team, Mass balance of the Antarctic Ice Sheet from 1992 to 2017. *Nature* **558**, 219–222 (2018).

6. S. G. Purkey, G. C. Johnson, Warming of Global Abyssal and Deep Southern Ocean Waters between the 1990s and 2000s: Contributions to global heat and sea level rise budgets. *J. Climate* **23**, 6336–6351 (2010).

7. A. H. Orsi, S. S. Jacobs, A. L. Gordon, M. Visbeck, Cooling and ventilating the abyssal ocean. *Geophys. Res. Lett.* **28**, 2923–2926 (2001).

8. P. W. Boyd, M. J. Ellwood, The biogeochemical cycle of iron in the ocean. *Nat. Geosci.* **3**, 675–682 (2010).

9. K. O. Forsch, L. Hahn-Woernle, R. M. Sherrell, V. J. Roccanova, K. Bu, D. Burdige, M. Vernet, K. A. Barbeau, Seasonal dispersal of fjord meltwaters as an important source of iron and manganese to coastal Antarctic phytoplankton. *Biogeosciences* **18**, 6349–6375 (2021).

10. O. Schofield, H. W. Ducklow, D. G. Martinson, M. P. Meredith, M. A. Moline, W. R. Fraser, How do polar marine ecosystems respond to rapid climate change? *Science* **328**, 1520–1523 (2010).

11. D. K. A. Barnes, A. Fleming, C. J. Sands, M. L. Quartino, D. Deregibus, Icebergs, sea ice, blue carbon and Antarctic climate feedbacks. *Phil. Trans. R. Soc. A.* **376**, 20170176 (2018).

12. K. O. Buesseler, A. M. P. McDonnell, O. M. E. Schofield, D. K. Steinberg, H. W. Ducklow, High particle export over the continental shelf of the west Antarctic Peninsula. *Geophys. Res. Lett.* **37**, L22606 (2010).

13. A. A. Petty, P. R. Holland, D. L. Feltham, Sea ice and the ocean mixed layer over the Antarctic shelf seas. *Cryosphere* **8**, 761–783 (2014).

14. S. L. Howard, J. Hyatt, L. Padman, Mixing in the pycnocline over the western Antarctic Peninsula shelf during Southern Ocean GLOBEC. *Deep Sea Res. Part II Top. Stud. Oceanogr.* **51**, 1965–1979 (2004).

15. J. A. Brearley, M. P. Meredith, A. C. Naveira Garabato, H. J. Venables, M. E. Inall, Controls on turbulent mixing on the West Antarctic Peninsula shelf. *Deep-Sea Res. II Top. Stud. Oceanogr.* **139**, 18–30 (2017).

16. T. P. Rippeth, Mixing in seasonally stratified shelf seas: A shifting paradigm. *Philos. Trans. A Math. Phys. Eng. Sci.* **363**, 2837–2854 (2005).

17. H. Burchard, T. P. Rippeth, Generation of bulk shear spikes in shallow stratified tidal seas. *J. Phys. Oceanogr.* **39**, 969–985 (2009).

18. M. E. Inall, J. A. Brearley, S. F. Henley, A. D. Fraser, S. Reed, Landfast ice controls on turbulence in Antarctic coastal seas. *J. Geophys. Res. Oceans* **127**, e2021JC017963 (2022).

19. A. J. Cook, A. J. Fox, D. G. Vaughan, J. G. Ferrigno, Retreating glacier fronts on the antarctic peninsula over the past half-century. *Science* **308**, 541–544 (2005).

20. I. Vaňková, D. M. Holland, Calving signature in ocean waves at Helheim Glacier and Sermilik Fjord, East Greenland. *J. Phys. Oceanogr.* **46**, 2925–2941 (2016).

21. J. M. Amundson, M. Truffer, M. P. Lüthi, M. Fahnestock, M. West, R. J. Motyka, Glacier, fjord, and seismic response to recent large calving events, Jakobshavn Isbræ, Greenland. *Geophys. Res. Lett.* **35**, L22501 (2008).

22. M. P. Lüthi, A. Vieli, Multi-method observation and analysis of a tsunami caused by glacier calving. *Cryosphere* **10**, 995–1002 (2016).

23. C. Baumhoer, A. Dietz, S. Dech, C. Kuenzer, Remote sensing of Antarctic glacier and ice-shelf front dynamics—A review. *Remote Sens.* **10**, 1445 (2018).

24. R. Mottram, N. Hansen, C. Kittel, J. M. van Wessem, C. Agosta, C. Amory, F. Boberg, W. J. van de Berg, X. Fettweis, A. Gossart, N. P. M. van Lipzig, E. van Meijgaard, A. Orr, T. Phillips, S. Webster, S. B. Simonsen, N. Souverijns, What is the surface mass balance of Antarctica? An intercomparison of regional climate model estimates. *Cryosphere* **15**, 3751–3784 (2021).

25. Y. Liu, J. C. Moore, X. Cheng, R. M. Gladstone, J. N. Bassis, H. Liu, J. Wen, F. Hui, Ocean-driven thinning enhances iceberg calving and retreat of Antarctic ice shelves. *Proc. Natl. Acad. Sci. U.S.A.* **112**, 3263–3268 (2015).

26. M. A. Depoorter, J. L. Bamber, J. A. Griggs, J. T. M. Lenaerts, S. R. M. Lightenberg, M. R. van den Broeke, G. Moholdt, Calving fluxes and basal melt rates of Antarctic ice shelves. *Nature* **502**, 89–92 (2013).

27. J. Todd, P. Christoffersen, T. Zwinger, P. Råback, D. I. Benn, Sensitivity of a calving glacier to ice–ocean interactions under climate change: New insights from a 3-D full-Stokes model. *Cryosphere* **13**, 1681–1694 (2019).

28. M. Schaefer, H. Machguth, M. Falvey, G. Casassa, E. Rignot, Quantifying mass balance processes on the Southern Patagonia Icefield. *Cryosphere* **9**, 25–35 (2015).

29. W. Kochtitzky, L. Copland, Retreat of Northern Hemisphere Marine-Terminating Glaciers, 2000–2020. *Geophys. Res. Lett.* **49**, e2021GL096501 (2022).

30. J. Tournadre, N. Bouhier, F. Girard-Ardhuin, F. Remy, Antarctic icebergs distributions 1992-2014. *J. Geophys. Res.* **121**, 327, 349 (2016).

31. A. J. Fox, A. Paul, R. Cooper, Measured properties of the Antarctic ice sheet derived from the SCAR Antarctic digital database. *Polar Rec.* **30**, 201–206 (1994).

32. D. G. Vaughan, J. C. Comiso, I. Allison, J. Carrasco, G. Kaser, R. Kwok, P. Mote, T. Murray, F. Paul, J. Ren, E. Rignot, O. Solomina, T. Zhang, Observations: Cryosphere, in *Climate Change 2013: The Physical Science Basis. Contribution of Working Group I to the Fifth Assessment Report of the Intergovernmental Panel on Climate Change*, T. F. Stocker, D. Qin, G.-K. Plattner, M. Tignor, S. K. Allen, J. Boschung, A. Nauels, Y. Xia, V. Bex, P. M. Midgley, Eds. (Cambridge Univ. Press, 2013), p. 66.

33. M. van den Broeke, J. Bamber, J. Ettema, E. Rignot, E. Schrama, W. J. van de Berg, E. van Meijgaard, I. Velicogna, B. Wouters, Partitioning recent Greenland mass loss. *Science* **326**, 984–986 (2009).

34. C. S. Andresen, F. Straneo, M. H. Ribergaard, A. A. Bjørk, T. J. Andersen, A. Kuijpers, N. Nørgaard-Pedersen, K. H. Kjær, F. Schjøth, K. Weckström, A. P. Ahlstrøm, Rapid response of Helheim Glacier in Greenland to climate variability over the past century. *Nat. Geosci.* **5**, 37–41 (2012).

35. A. Luckman, D. I. Benn, F. Cottier, S. Bevan, F. Nilsen, M. Inall, Calving rates at tidewater glaciers vary strongly with ocean temperature. *Nat. Commun.* **6**, 8566 (2015).

36. J. Turner, H. Lu, I. White, J. C. King, T. Phillips, J. S. Hosking, T. J. Bracegirdle, G. J. Marshall, R. Mulvaney, P. Deb, Absence of 21st century warming on Antarctic Peninsula consistent with natural variability. *Nature* **535**, 411–415 (2016).

37. J. Turner, S. R. Colwell, G. J. Marshall, T. A. Lachlan-Cope, A. M. Carleton, P. D. Jones, V. Lagun, P. A. Reid, S. Iagovinka, Antarctic climate change during the last 50 years. *Int. J. Climatol.* **25**, 279–294 (2005).

38. I. M. Howat, C. Porter, B. E. Smith, M.-J. Noh, P. Morin, The reference elevation model of Antarctica. *Cryosphere* **13**, 665–674 (2019).

39. D. I. Benn, J. A. Åström, Calving glaciers and ice shelves. *Adv. Phys: X* **3**, 1513819 (2018).

40. E. Firing, J. M. Hummon, Shipboard ADCP measurement, in *The GO-SHIP Repeat Hydrography Manual: A Collection of Expert Reports and Guidelines*. IOC –SCOR International Ocean Carbon Coordination Project, Report 14 (2010).

41. T. E. Ryan, R. A. Downie, R. J. Kloser, G. Keith, Reducing bias due to noise and attenuation in open-ocean echo integration data. *ICES J. Mar. Sci.* **72**, 2482–2493 (2015).

42. A. De Robertis, I. Higginbottom, A post-processing technique to estimate the signal-to-noise ratio and remove echosounder background noise. *ICES J. Mar. Sci.* **64**, 1282–1291 (2007).

43. A. M. Mair, P. G. Fernandes, A. Lebourges-Dhaussy, A. S. Brierley, An investigation into the zooplankton composition of a prominent 38-kHz scattering layer in the North Sea. *J. Plankton Res.* **27**, 623–633 (2005).

44. A. C. Lavery, P. H. Wiebe, T. K. Stanton, G. L. Lawson, M. C. Benfield, N. Copley, Determining dominant scatterers of sound in mixed zooplankton populations. *J. Acoust. Soc. Am.* **122**, 3304–3326 (2007).

45. D. Thomson, Spectrum estimation and harmonic analysis. *Proc. IEEE* **70**, 1055–1096 (1982).

46. D. B. Percival, A. T. Walden, *Spectral Analysis for Physical Applications* (Cambridge Univ. Press, 1993).

47. M. Inall, D. Aleynik, T. Boyd, M. Palmer, J. Sharples, Internal tide coherence and decay over a wide shelf sea. *Geophys. Res. Lett.* **38**, L23607 (2011).

48. T. R. Osborn, Estimates of the local rate of vertical diffusion from dissipation measurements. *J. Phys. Oceanogr.* **10**, 83–89 (1980).

49. J. Marshall, A. Adcroft, C. Hill, L. Perelman, C. Heisey, A finite-volume, incompressible Navier Stokes model for studies of the ocean on parallel computers. *J. Geophys. Res.,* **102**, 5753–5766 (1997).

50. J. Marshall, C. Hill, L. Perelman, A. Adcroft, Hydrostatic, quasi-hydrostatic, and non-hydrostatic ocean modeling. *J. Geophys. Res. Oceans* **102**, 5733–5752 (1997).

51. D. R. Jackett, T. J. McDougall, A neutral density variable for the world's oceans. *J. Phys. Oceanogr.* **27**, 237–263 (1997).

52. A. Adcroft, J. M. Campin, Rescaled height coordinates for accurate representation of free-surface flows in ocean circulation models. *Ocean Model.* **7**, 269–284 (2004).

53. A. Adcroft, C. Hill, J. Marshall, Representation of topography by shaved cells in a height coordinate ocean model. *J. Phys. Oceanogr.* **125**, 2293–2315 (1997).

54. V. Daru, C. Tenaud, High order one-step monotonicity-preserving schemes for unsteady compressible flow calculations. *J. Comp. Phys.* **193**, 563–594 (2004).

55. C. Hill, D. Ferreira, J. M. Campin, J. Marshall, R. Abernathey, N. Barrier, Controlling spurious diapycnal mixing in eddy-resolving height-coordinate ocean models – Insights from virtual deliberate tracer release experiments. *Ocean Model.* **45–46**, 14–26 (2012).

56. R. C. Pacanowski, S. G. Philander, Parameterization of vertical mixing in numerical models of tropical oceans. *J. Phys. Oceanogr.* **11**, 1443–1451 (1981).

57. G. D. Egbert, R. D. Ray, Significant dissipation of tidal energy in the deep ocean inferred from satellite altimeter data. *Nature* **405**, 775–778 (2000).

58. J. A. M. Green, J. Nycander, A comparison of tidal conversion parameterizations for tidal models. *J. Phys. Oceanogr.* **43**, 104–119 (2013).

59. M. E. Inall, M. Toberman, J. A. Polton, M. R. Palmer, J. A. M. Green, T. P. Rippeth, Shelf seas baroclinic energy loss: Pycnocline mixing and bottom boundary layer dissipation. *J. Geophys. Res. Oceans* **126**, e2020JC016528 (2021).

60. L. Padman, S. Y. Erofeeva, H. A. Fricker, Improving Antarctic tide models by assimilation of ICES at laser altimetry over ice shelves. *Geophys. Res. Lett.* **35**, L22504 (2008).

61. P. Weatherall, K. M. Marks, M. Jakobsson, T. Schmitt, S. Tani, J. E. Arndt, M. Rovere, D. Chayes, V. Ferrini, R. Wigley, A new digital bathymetric model of the world's oceans. *Earth Space Sci.* **2**, 331–345 (2015).

62. M. R. Mazloff, P. Heimbach, C. Wunsch, An eddy-permitting southern ocean state estimate. *J. Phys. Oceanogr.* **40**, 880–899 (2010).

63. J. H. Simpson, D. G. Bowers, The role of tidal stirring in controlling the seasonal heat cycle in shelf seas. *Ann. Geophys.* **2**, 411–416 (1984).

64. J. H. Simpson, W. R. Crawford, T. P. Rippeth, A. R. Campbell, J. V. S. Cheok, The vertical structure of turbulent dissipation in shelf seas. *J. Phys. Oceanogr.* **26**, 1579–1590 (1996).

65. M. H. Alford, Revisiting near-inertial wind work: Slab models, relative stress, and mixed layer deepening. *J. Phys. Oceanogr.* **50**, 3141–3156 (2020).

66. J. F. Price, R. A. Weller, R. Pinkel, Diurnal cycling: Observations and models of the upper ocean response to diurnal heating, cooling, and wind mixing. *J. Geophys. Res.* **91**, 8411 (1986).

67. H. Hersbach, B. Bell, P. Berrisford, S. Hirahara, A. Horányi, J. Muñoz-Sabater, J. Nicolas, C. Peubey, R. Radu, D. Schepers, A. Simmons, C. Soci, S. Abdalla, X. Abellan, G. Balsamo, P. Bechtold, G. Biavati, J. Bidlot, M. Bonavita, G. Chiara, P. Dahlgren, D. Dee, M. Diamantakis, R. Dragani, J. Flemming, R. Forbes, M. Fuentes, A. Geer, L. Haimberger, S.

Healy, R. J. Hogan, E. Hólm, M. Janisková, S. Keeley, P. Laloyaux, P. Lopez, C. Lupu, G. Radnoti, P. Rosnay, I. Rozum, F. Vamborg, S. Villaume, J. Thépaut, The ERA5 global reanalysis. *Q. J. R. Meteorol. Soc.* **146**, 1999–2049 (2020).

68. H. J. Venables, M. P. Meredith, J. A. Brearley, Modification of deep waters in Marguerite Bay, western Antarctic Peninsula, caused by topographic overflows. *Deep-Sea Res. II Top. Stud. Oceanogr.* **139**, 9–17 (2017).

69. F. Straneo, R. G. Curry, D. A. Sutherland, G. S. Hamilton, C. Cenedese, K. Vage, L. A. Stearns, Impact of fjord dynamics and glacial runoff on the circulation near Helheim Glacier. *Nat. Geosci.* **4**, 322–327 (2011).

70. D. A. Slater, F. Straneo, S. B. Das, C. G. Richards, T. J. W. Wagner, P. W. Nienow, Localized plumes drive front-wide ocean melting of a Greenlandic tidewater glacier. *Geophys. Res. Lett.* **45**, 12350–12358 (2018).

71. J. M. Lea, The Google Earth Engine Digitisation Tool (GEEDiT) and the Margin change Quantification Tool (MaQiT): Simple tools for the rapid mapping and quantification of changing Earth surface margins. *Earth Surf. Dyn.* **6**, 551–561 (2018).

72. T. Moon, I. Joughin, Changes in ice front position on Greenland's outlet glaciers from 1992 to 2007. *J. Geophys. Res.,* **113**, F02022 (2008).

73. T. Moon, I. Joughin, B. Smith, M. R. van den Broeke, W. J. van de Berg, B. Noël, M. Usher, Distinct patterns of seasonal Greenland glacier velocity. *Geophys. Res. Lett.* **41**, 7209–7216 (2014).

74. C. Amante, B. W. Eakins, ETOPO1 1 Arc-Minute Global Relief Model: Procedures, data sources and analysis (NESDIS NGDC-24, NOAA National Geophysical Data Center); 10.7289/V5C8276M (2009).

75. H. G. Gade, Melting of ice in sea water: A primitive model with application to the Antarctic ice shelf and icebergs. *J. Phys. Oceanogr.* **9**, 189–198 (1979).
